# Characterization of subclinical ZIKV infection in immune-competent guinea pigs and mice

**DOI:** 10.1099/jgv.0.001641

**Published:** 2021-08-19

**Authors:** Joseph A. Westrich, Erin E. McNulty, Marisa J. Edmonds, Amy V. Nalls, Megan R. Miller, Brian D. Foy, Joel Rovnak, Rushika Perera, Candace K. Mathiason

**Affiliations:** ^1^​ Department of Microbiology, Immunology, and Pathology, Colorado State University, Fort Collins, Colorado, USA

**Keywords:** animal model, fetal transmission, placenta, Zika virus

## Abstract

An infectious agent’s pathogenic and transmission potential is heavily influenced by early events during the asymptomatic or subclinical phase of disease. During this phase, the presence of infectious agent may be relatively low. An important example of this is Zika virus (ZIKV), which can cross the placenta and infect the foetus, even in mothers with subclinical infections. These subclinical infections represent roughly 80 % of all human infections. Initial ZIKV pathogenesis studies were performed in type I interferon receptor (IFNAR) knockout mice. Blunting the interferon response resulted in robust infectivity, and increased the utility of mice to model ZIKV infections. However, due to the removal of the interferon response, the use of these models impedes full characterization of immune responses to ZIKV-related pathologies. Moreover, IFNAR-deficient models represent severe disease whereas less is known regarding subclinical infections. Investigation of the anti-viral immune response elicited at the maternal-foetal interface is critical to fully understand mechanisms involved in foetal infection, foetal development, and disease processes recognized to occur during subclinical maternal infections. Thus, immunocompetent experimental models that recapitulate natural infections are needed. We have established subclinical intravaginal ZIKV infections in mice and guinea pigs. We found that these infections resulted in: the presence of both ZIKV RNA transcripts and infectious virus in maternal and placental tissues, establishment of foetal infections and ZIKV-mediated CXCL10 expression. These models will aid in discerning the mechanisms of subclinical ZIKV mother-to-offspring transmission, and by extension can be used to investigate other maternal infections that impact foetal development.

## Importance

Zika virus (ZIKV) infection of pregnant women is associated with offspring congenital abnormalities. Although roughly 80 % of ZIKV infections are asymptomatic, these infections have been shown to be as detrimental to foetal health. Although severe ZIKV disease pathology has been well defined using type I interferon receptor knockout mice, reliable small animal models to recapitulate subclinical disease, in an immune-competent host, remain under investigated. Here, we report subclinical ZIKV infections in pregnant guinea pigs and mice. In these animals, we found ZIKV in maternal and foetal tissues, as well as increased CXCL10 expression in infected tissues, as is observed in human cases. These findings provide a valuable tool to understand the anti-viral immune response against ZIKV that occurs at the maternal-foetal interface and to evaluate the impact of subclinical ZIKV infection on foetal health.

## Introduction

ZIKV infection of pregnant women has been causally associated with congenital abnormalities and foetal loss [1]. ZIKV has been shown to cross the placental structure and infect the foetus, even in mothers with subclinical ZIKV infections [2]. Importantly, although 80 % of ZIKV infections are asymptomatic, these subclinical infections were just as detrimental to the foetal development [2–4]. In addition to severe anatomical abnormalities, ZIKV is also responsible for more subtle and prolonged negative effects on offspring development [5]. Although the American ZIKV epidemic has passed, ZIKV continues to circulate around the world [6]. And as predicting future outbreaks is not yet feasible, the expanded geographical distribution of competent vectors with ability to transmit this flavivirus [7] necessitates continued exploration of the mechanisms associated with asymptomatic ZIKV infections. To this end, experimental models are needed to recapitulate human disease and the anti-viral immune response elicited by the presence of ZIKV at the maternal-foetal interface. The most utilized model to study ZIKV pathology is an immune-deficient mouse [8]. This model has contributed greatly to the knowledge of the pathogenesis of the virus, yet, it is not ideal to study the immunological effects of the virus on the foetus. Several studies have suggested that immune-competent mice may be susceptible to low-level infection, but they have not been thoroughly characterized in this regard [8]. Another rodent species, guinea pigs, have also previously been shown to be susceptible to ZIKV infection [9–12]. Like the studies with wild-type mice, these infections appear to be associated with low levels of virus replication with minimal, if any, clinical signs.

Here we report the characterization of subclinical ZIKV infection in two immune-competent pregnancy models. We have found that intravaginal, but not subcutaneous, route of inoculation in early/mid-term pregnancy results in detectable and infectious virus in wild-type mouse and Hartley guinea pig dams for up to a week post-inoculation. Although ZIKV was detected, no outward clinical signs were evident in the infected animals. ZIKV infection was further shown to be harboured long term in placental tissue, although no clear anatomical impact was observed in foetuses of these animals.

## Methods

### Mice and guinea pigs

Breeding pairs of C57BL/6J and B6.129S2-Ifnar1tm1Agt/Mmjax (interferon ⍺/ß receptor knockout, IFNAR1^-/-^) were obtained from Jackson Laboratory and bred in house. Mice were genotyped according to recommended protocols (https://www.jax.org/ Protocol ID 29023). For non-pregnancy studies, mice of both sexes were used between 6 and 9 weeks of age. For pregnancy studies, female mice were co-housed overnight with a male, which was removed the following day. Pregnancy was determined by monitoring mouse weight until E12 in which mice with greater than 2 g weight gain were predicted to be pregnant. Hartley guinea pigs were purchased from Elm Hill Laboratory. For pregnancy studies, 7-week-old females were received and allowed to acclimate for 7 days prior to experimental exposure. For pregnant guinea pig studies, female guinea pigs were co-housed with a male and pregnancy was determined by monitoring dam weight until E21 in which 30 g weight gain over non-pregnant controls and/or palpitations were used to predict pregnancy.

### Cell culture and plaque forming assay

African Green Monkey kidney cells (Vero) and Human placental cell line (JEG-3) were purchased from the American Type Culture Collection (ATCC; Manassas, VA). Cells were maintained at 37 °C with 5 % CO2 in Dulbecco’s modified Eagle medium (DMEM, Gibco) (Vero) or Eagle's minimum essential medium (MEM, Gibco) (JEG-3) supplemented with 10 % foetal bovine serum (FBS, Gibco), 2 mm l-glutamine, 1.5 g l^−1^ sodium bicarbonate, 100 U ml^−1^ penicillin, 100 µg ml^−1^ streptomycin. Infectious titre of ZIKV was determined by plaque assay. Briefly, Vero cells (2×10^5^ cells/well) were plated in 12-well plates and inoculated with serially diluted viruses and overlaid with a 2 % carboxymetholose overlay. Five days post-inoculation, the cells were fixed with 10 % formaldehyde solution in phosphate-buffered saline, stained with a methylene blue solution, clarified and quantified.

### Zika virus propagation

ZIKV strain PRVABC59 (ZIKV-PR; GenBank: KU501215), was a gift from Aaron Brault (CDC, Ft. Collins, CO) and was originally isolated in 2015 from a traveller to Puerto Rico. Virus was amplified three rounds in Vero cells. ZIKV was quantified by plaque-forming assays (described above). The virus stock is routinely used up to passage 3 without additional sequencing.

### Animal infection studies

Animals were anesthetized using isoflurane gas. When the animal was non-responsive to stimuli, it received the inoculation by either subcutaneous or intravaginal route. For the subcutaneous route, animals were injected using a 1 ml tuberculin syringe with a 28-gauge needle. A bolus of inoculum or PBS (for mock-infected animals) was injected just under the skin at the nape of the neck. For intravaginal infections, the anesthetized animal was laid in a supine position and a calcium alginate swab (Fisher Scientific, Cat. No. 22-029-501), was inserted into the vaginal canal to disrupt the mucosal layer. Immediately following, the volume of inoculum, or PBS control, was administered to the vaginal canal by either a p20 (mouse) or p200 (guinea pig) pipette. In both inoculation routes, animals were allowed to recover from anaesthesia, which was ensured by independent mobility. Animal weight was monitored throughout the time course of infection. Intermittent blood draws were performed for virus detection. Animals that lost more the 20 % of starting weight were euthanized by CO_2_ inhalation followed by secondary cervical dislocation. At the end of study, all animals were humanely euthanized and tissues were collected for further analysis. Foetal skull area was determined by lateral and tangential measurement of the skull using electronic calliper (Fisherbrand). All methods and study parameters were performed in accordance to the Colorado State University IACUC under the approved protocol (Protocol No. 19-9013A, #1055).

### Reverse transcriptase-quantitative PCR (RT-qPCR)

Total RNA was extracted using either a QIAamp Viral mRNA minikit or RNeasy Mini kit (Qiagen) with on-column DNase digestion using the RNase-free DNase (Qiagen) according to the suppliers’ instructions. First-strand cDNA was synthesized using a Transcriptor First Strand cDNA Synthesis Kit (Roche) from 1 µg of total RNA. Real-time PCR was performed in a 20 µl reaction mixture containing 10 µl of FastStart Universal SYBR green master (Rox, Roche Applied Science), 0.5 µm of each primer, 5 µl of cDNA template, and nuclease-free water using the Bio-Rad CFT Connect real-time system. Primers used in this study are described in Table 1. *Cxcl10* expression data were normalized to Actin mRNA. ZIKV quantification was determined by concurrent running of a known quantity of ZIKV plasmid [13]. Based on these results our limit of detection is roughly 60 copies per mg.

**Table 1.** RT-qPCR primers used in this study

Primer target	Forward 5′ −3′	Reverse 5′−3′
PRVABC59 ZIKV	AAGTACACATACCAAAACAAAGTGGT	TCCGCTCCCCCTTTGGTCTTG
Mouse actin	TCACCCACACTGTGCCCATCTA	TGAGGTAGTCAGTCAGGTCCCG
Mouse cxcl10	CTTCTGAAAGGTGACCAGCC	GTCGCACCTCCACATAGCTT
Guinea pig actin	CCAACTGGGACGACATGGAG	CGTAGCCCTCGTAGATGGGC
Guinea pig cxcl10	GCCACAATGAAAATGAATG	CTGCTTTCAGTAAATTCTTAATG
Human ACTIN	TCACCCACACTGTGCCCATCTA	TGAGGTAGTCAGTCAGGTCCCG
Human CXCL10	GAAATTATTCCTGCAAGCCAATTT	TCACCCTTCTTTTTCATTGTAGCA

### Immunohistochemistry (IHC)

PLP fixed mouse and guinea pig placentas and foetuses were paraffin-embedded and 5 µm tissue sections were cut and mounted on glass positive charge slides. Tissues were deparaffinized in a 65 °C oven followed by successive xylene immersions (100%), and rehydrated through graded ethanol washes (100 % × 2, 95 % × 2 and 70 % at 5 min per wash). Tissues were then subjected to hydrated autoclaving using an automated antigen-retrieval system 2100-Retriever (Prestige Medical) and a citrate buffer (0.01M sodium citrate, 0.05 % tween 20, pH 6) for 30 min. Samples were then blocked with 3 % H2O2 (20 min) followed by a proprietary protein block (TNB, PerkinElmer Life and Analytical Sciences) (30 min) and stained with unconjugated Anti-NS1 at 1 : 500 for mice and neat for guinea pigs (Abcam, ab214337) (overnight at 4 °C). Detection was completed using HRP-conjugated anti-mouse secondary antibody (Envision+, Dako) (30 min) and AEC substrate chromogen (Dako) (3 min for mouse tissues, 10 min for guinea pig). Tissues were then counterstained with Meyer’s hematoxylin (Dako) (2 min), followed by 0.1 % calcium bicarbonate bluing reagent (5 min) and coverslipped with aqueous mounting media (Dako). For haematoxylin and eosin stain (H&E), tissues were stained with Meyer’s hematoxylin (described above) and counterstained with Eosin (novaultra) for 30 s and washed 3 × in 95 % ethanol, followed by two immersions in xylene for 10 mins each and coverslipped with xylene based mounting media (Permount).

### Statistical analysis

Student’s *t*-test and two-way ANOVA were used to calculate significance for comparison of two matched groups and three or more unmatched groups, respectively using Prism 8 (GraphPad). Results were considered statistically significant at a *P*-value of less than 0.05.

## Results

### Subcutaneous ZIKV inoculation does not establish infection in non-pregnant immune-competent animals

#### IFNAR 1-/- mouse subcutaneous inoculation

**Fig. 1.**
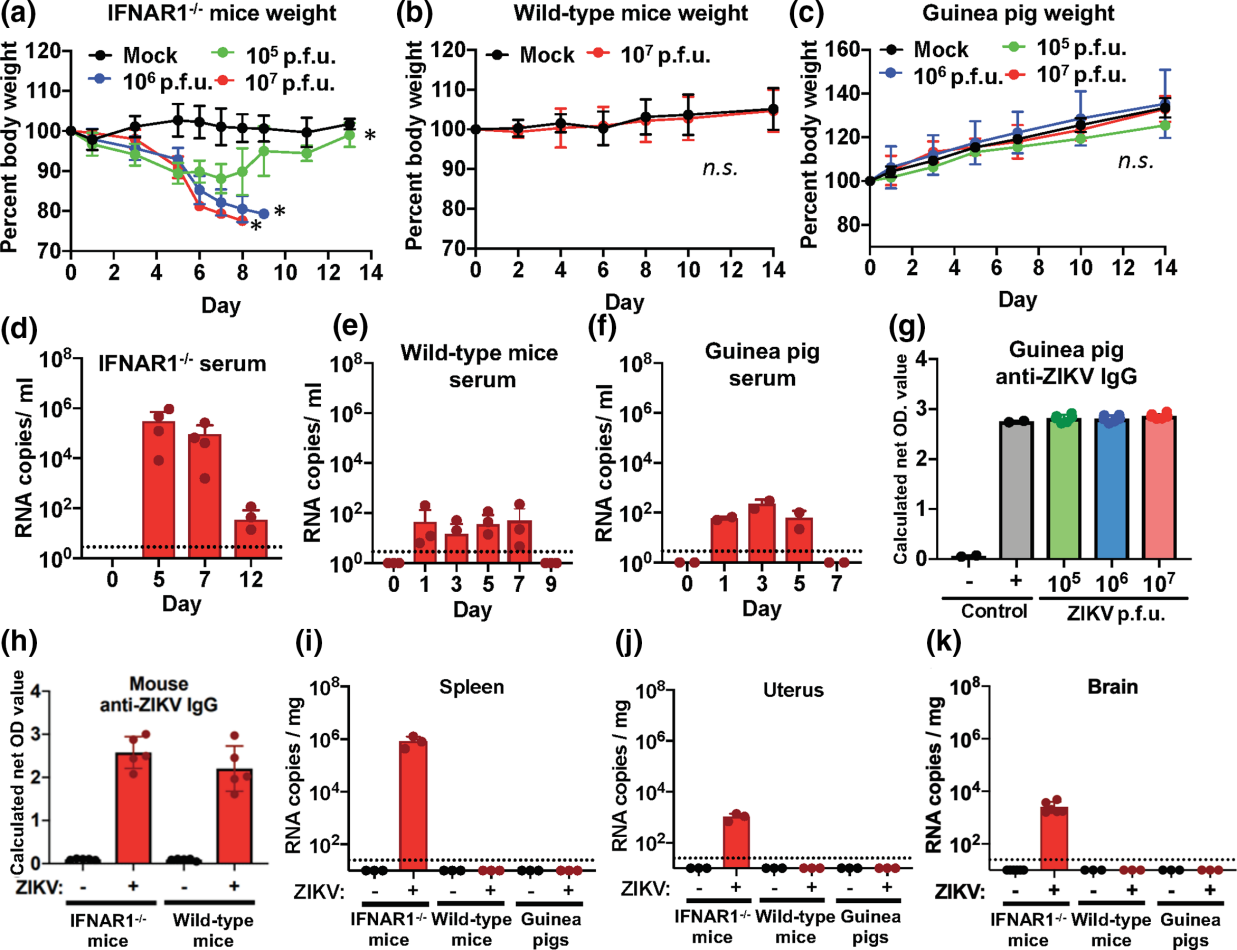
Subcutaneous ZIKV inoculation exhibits minimal disease in immunocompetent animals. IFNAR1^-/-^ (a), wild-type mice (b) and guinea pigs (c) were inoculated with three titres of PRVABC59 ZIKV subcutaneously and monitored for weight loss as compared to their pre-inoculation weight over 2 weeks. Blood was drawn at regular intervals and ZIKV viral RNA loads was determined by RT-qPCR for IFNAR1^-/-^ (d), wild-type mice (e) and guinea pigs (f). Guinea pig, wild-type mouse, and IFNAR1^-/-^ serum was evaluated for anti-ZIKV antibodies using an ZIKV specific ELISA at 14 days p.i. (g and h). Spleens (i), uterus (j) and brains (k) of the animals were evaluated at 14 days p.i. for ZIKV viral burden by RT-qPCR. Statistical differences in weight loss were determined by two-way ANOVA and * denotes significance of *P* <0.05. *n.s.* denotes non-significant differences. Data shown is representative of two experimental replicates (cohorts range from *n*=3–10/group for mice and *n*=3–4/group for guinea pigs).

Previous studies have shown IFNAR1^-/-^ mice to be susceptible to clinically apparent ZIKV infection through subcutaneous route of inoculation [8]. To ensure our methods of inoculation were efficacious, we inoculated IFNAR1^-/-^ mice (*n*=3/group) with three different titres of PRVABC59 ZIKV virus, 10^5^, 10^6^, and 10^7^ p.f.u. In agreement with published findings, we found that IFNAR1^-/-^ mice, inoculated with ZIKV subcutaneously, developed a clinical sign of weight loss in a dose-dependent manner (Fig. 1a). Animals inoculated with higher titre (10^6^ and 10^7^ p.f.u.) virus reached end-point criteria by day 8–9 post-inoculation (p.i.), but mice injected with 10^5^ viruses ultimately were able to control the virus, and recover from the infection. To determine if ZIKV disseminated from the site of inoculation, we evaluated viral burden in spleen, serum, brain and reproductive tissues by RT-qPCR (Fig. 1d–k). Robust viral RNA loads were evident in all tissue types of IFNAR1^-/-^ mice. These data show subcutaneous inoculation is a viable route of infection for ZIKV, and IFNAR1^-/-^ mice are susceptible to clinical ZIKV infection through this route.

#### Wildtype C57BL/6J mouse subcutaneous inoculation

We next determined if immune-competent, wild-type C57BL/6J mice were also susceptible to ZIKV through the subcutaneous route of inoculation. Wild-type mice have been previously shown to be more resistant to infection. This is understood to be due, in part, to the antagonism of STAT2, a key signalling protein in the interferon pathway, by the ZIKV viral proteins and is shown to be highly species specific [14]. Although this certainly may be critical for robust clinical infections, given the findings that a multitude of species can be infected with ZIKV, it may not be essential for all, particularly mild infections [15, 16]. Given the inherent resistance of wild-type mice, we injected only with the highest titre, 10^7^ p.f.u. of PRVABC59 ZIKV (*n*=10/group). Animals weight was monitored for 2 weeks p.i. and observed no clear loss of weight in the infected animals over mock-injected controls (Fig. 1b). Evaluation of spleen, reproductive and brain tissues showed no evidence for ZIKV infection (Fig. 1i–k). Although no virus was detectable in the tissues of the animals, low-level transient viral RNA loads were observed for up to 7 days p.i. and was thereafter undetectable (Fig. 1e). In addition to the low-level transient viral RNA loads detected in the serum, an anti-ZIKV IgG response was observed in both wild-type and IFNAR^-/-^ mice at 9 days p.i. (Fig. 1h). Combined, these data suggest that immune-competent mice are not susceptible to clinical or disseminated ZIKV infection through subcutaneous route of inoculation, unlike findings in IFNAR1^-/-^ mice. Interestingly, with the exception of cases with severe disease pathology, transient viral loads are observed in many human ZIKV cases as well [17, 18].

#### Guinea pig subcutaneous inoculation

Guinea pigs are another small animal model that has been shown to be susceptible to ZIKV infection [9–12]. Given that guinea pigs are more similar to humans immunologically and developmentally as compared to mice, they represent a promising model to recapitulate human ZIKV disease [19, 20]. To determine if guinea pigs are susceptible to subcutaneous ZIKV infection, we inoculated cohorts (*n*=3/group) with three different doses of PRVABC59 ZIKV virus, 10^5^, 10^6^ and 10^7^ p.f.u. Monitoring the guinea pigs to day 35 we observed no significant change in weight as compared to mock-injected controls (Fig. 1c). Although the animals mounted a serological response to the inoculation (Fig. 1g), evaluation of the tissues at 14 days p.i. to detect ZIKV revealed no consistent detection of virus (Fig. 1i–k). Furthermore, as was observed in the wild-type mice, low-level transient viral RNA loads were detectable for up to 5 days p.i. and undetectable thereafter (Fig. 1f). These data suggest that, like the wild-type mice, immune-competent guinea pigs are not susceptible to disseminating ZIKV infection following subcutaneous inoculation. Although no accumulation of ZIKV was observed in tissues, the low level of circulating virus that was observed in the serum may have consequence in a model of pregnancy as the maternal blood would bring direct exposure of virus to the maternal-foetal interface.

#### Intravaginal inoculation establishes detectable ZIKV infection in immune-competent pregnant animals

ZIKV has been shown to be sexually transmitted [21]. Several studies have shown that intravaginal inoculation of ZIKV establishes local infection [22, 23]. In order to reproducibly infect virgin mice, the females must first be treated with depot medroxyprogesterone acetate (DMPA), to ensure the animals are in the diestrus phase of the estrous cycle. It is in this phase that the animals are most susceptible to infection [24]. Alternatively, pregnant animals are susceptible to ZIKV infection throughout pregnancy without the need for hormonal adjustment [25]. It has been shown that early- to mid-gestation is a particularly sensitive window for foetal development and pathogenic insults, thus we aimed to infect the animals within this window [26]. Mice were inoculated at embryonic day 12 (E12) to allow for complete placentation that occurs between E10 and E10.5; as it has been shown that when ZIKV infection occurs early in development (prior to complete placentation), the foetus succumbs to the infection, and is reabsorbed by the mother [27]. This study also demonstrated that after gestational age E12.5, the impact of ZIKV infection is reduced, suggesting a significant role for the placental barrier.

#### IFNAR 1^-/-^ mouse intravaginal inoculation

**Fig. 2.**
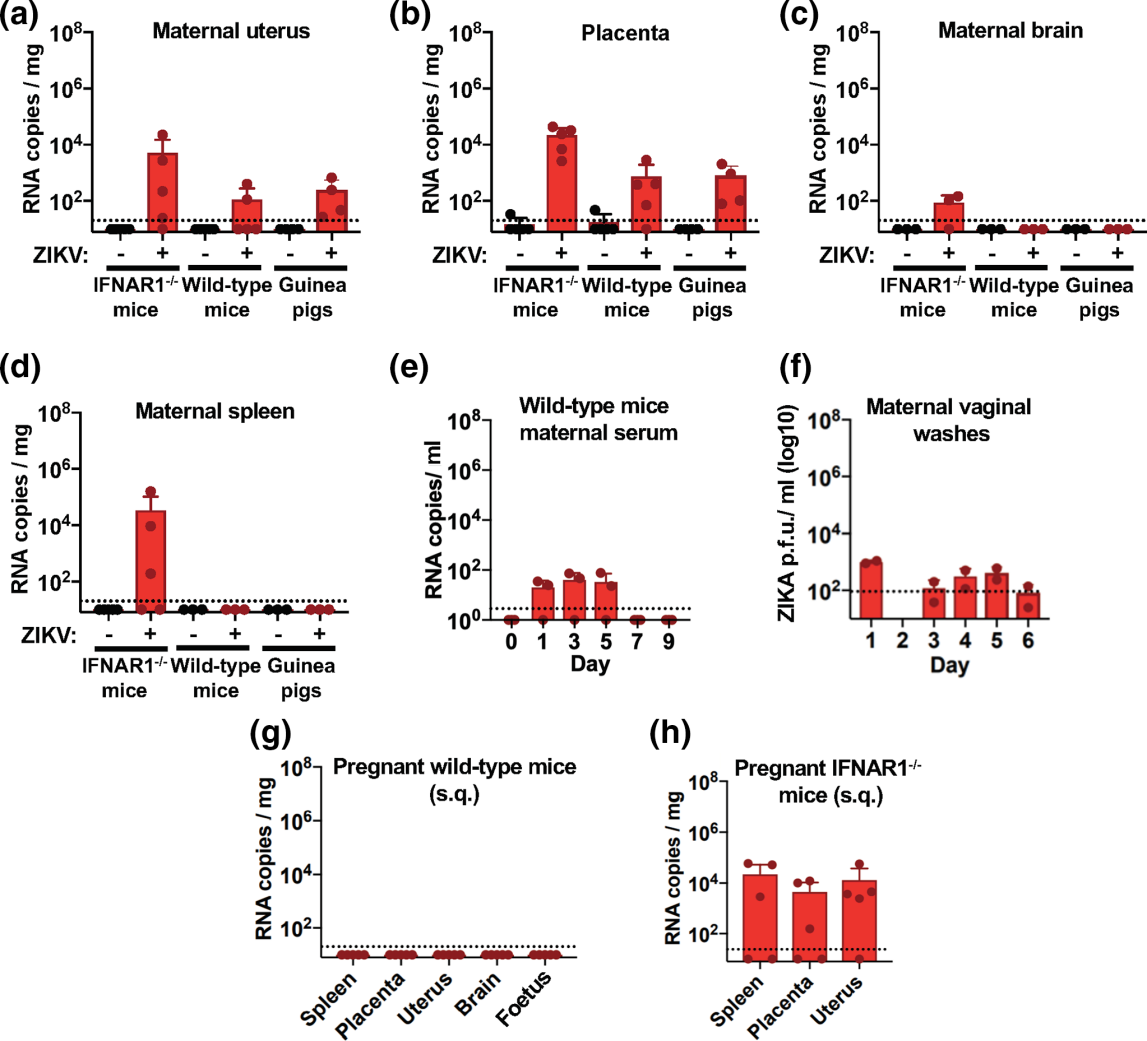
Intravaginal ZIKV inoculation leads to viral detection in maternal tissues. Pregnant IFNAR1^-/-^, wild-type mice, and guinea pigs were inoculated with 10^5^ p.f.u. (mice) or 10^7^ p.f.u. ZIKV (guinea pig) intravaginal at E12 (mice) or E21 (guinea pig). At 3 days p.i., animals were sacrificed and maternal uterus (a), placenta (b), brain (c), spleen (d) were processed for ZIKV assessment by RTq-PCR. Serum was harvested from wild-type mice via serial blood draws and evaluated for ZIKV by RT-qPCR (e). Vaginal washes collected from inoculated wild-type mice were evaluated for ZIKV presence by plaque-forming assay (shown is infectious virus detected in two of three animals evaluated) (f). A cohort of pregnant, wild-type (g) and IFNAR1^-/-^ (h) mice were inoculated subcuteanously and sacrificed at 3 days p.i. Maternal tissues were processed for ZIKV detection by RTq-PCR. Data shown is representative of three experimental replicates for mice and two experimental replicates for guinea pigs (cohorts range from *n*=3–5/group for mice and *n*=3–4/group for guinea pigs).

To evaluate if intravaginal route of inoculation is capable of establishing infection, we inoculated pregnant IFNAR1^-/-^ mice (*n*=5/group) at E12. Briefly, anesthetized, pregnant animals had their vaginal mucosa disrupted by a calcium-alginate swab. This was immediately followed by administration of 10^5^ p.f.u. PRVABC59 ZIKV directly in the vagina. At 3 days p.i., the animals were euthanized and viral RNA loads were determined for several tissues as was performed in the subcutaneous inoculated animals. We found viral RNA copies in the spleen, brain, placental and reproductive tissues of the pregnant dams (Fig. 2a–d). These data show that intravaginal inoculation of ZIKV is a viable method of virus infection and tissue dissemination in IFNAR1^-/-^ mice.

#### Wildtype C57BL/6J mouse intravaginal inoculation

As pregnancy establishes a unique immune environment, we next determined if pregnant, immune-competent mice were susceptible to ZIKV infection when intravaginally inoculated. As with the IFNAR1^-/-^ mice, we inoculated wild-type C57BL/6J pregnant mice (*n*=5/group) at E12. At 3 days p.i. we evaluated dam tissues for ZIKV infection. Evaluation of the tissues revealed low-level ZIKV titre in the serum, reproductive and placental tissues (Fig. 2a, b, e). Interestingly, we were not able to detect viral genomes in the maternal splenic or brain tissues, as was observed in the IFNAR1^-/-^ mice (Fig. 2c, d). To ensure we were not simply detecting the original inoculum we performed viral genome quantification and plaque-forming assays on vaginal washes to detect viral genomes and infectious virus, respectively. Infectious replicating virus was detected up to 6 days p.i. (Fig. 2f). These data suggest that intravaginal inoculation of pregnant wild-type animals establishes a localized ZIKV infection in reproductive tissue and low-level infection in these animals.

#### Hartley guinea pig intravaginal inoculation

Given the local infection observed in intravaginally inoculated pregnant wild-type mice, we sought to determine if pregnant guinea pigs were likewise susceptible to intravaginal ZIKV inoculation. Like the mice studies, and accounting for the longer gestation time of guinea pigs, we aimed to infect during the window of greatest susceptibility, i.e. ~21 days post-coitum (E21). This time point was chosen due to the level of development of the foetus and placenta in terms of susceptibility to infection without risk of pregnancy loss. At guinea pig gestational age E21 placentation and establishment of the placental blood supply is nearly complete [28], similar to E12 mice. A previous study inoculated pregnant guinea pig females between E18 and E21, and although viral detection was not observed on these animals, a possible reason for this could be the use of subcutaneous inoculation [10]. Thus, pregnant guinea pigs (*n*=4/group) were intravaginally inoculated 21 days post-coitum. At 3 days p.i. pregnant animals were euthanized and tissues were evaluated for ZIKV RNA. As was observed in the wild-type mice, ZIKV was detected at low levels in the reproductive and placental tissues (Fig. 2a, b), with no virus detected in splenic or brain tissues (Fig. 2c, d). Taken together, these data suggest that pregnant guinea pigs, like pregnant wild-type mice, establish a low-level ZIKV infection when inoculated intravaginally.

### Pregnancy in itself does not lead to increased wild-type C57BL/6J susceptibility to ZIKV after subcutaneous inoculation

To determine if pregnancy itself increases susceptibility to ZIKV infection we subcutaneously inoculated pregnant wild-type C57BL/6J mice (E12) with 10^7^ ZIKV (*n*=5/group). As wild-type C57BL/6J non-pregnant animals were previously shown not to be susceptible to persistent subcutaneous infection, we used the highest virus p.f.u. for inoculation. At 3 days p.i. pregnant subcutaneous inoculated wild-type mice were euthanized and evaluated for ZIKV RNA in tissues analysed previously. As with the subcutaneous inoculated non-pregnant wild-type mice we were unable to detect viral genomes in any of the tissues evaluated (Fig. 2g). We also evaluated a cohort of pregnant IFNAR1^-/-^ mice (*n*=5/group) inoculated at the same time during gestation and observed viral detection in spleen, uterus and placenta (Fig. 2h), further exemplifying the highly susceptible nature of IFNAR1^-/-^ mice to ZIKV infection. Given that the immune-competent wild-type mice in our study showed no detectable infection in reproductive tissues, in combination with a previous study [10] showing that subcutaneous inoculation of pregnant guinea pigs exhibited no impact on foetal development, we felt it would be an unnecessary use of the animals to repeat these studies in guinea pigs. These data suggest that pregnancy alone does not enhance susceptibility to ZIKV infection.

### Intravaginal ZIKV inoculation results in foetal infections in immune-competent mice and guinea pigs

We have detected ZIKV within the placental tissues of intravaginally infected animals (Fig. 2b). We next determined if the local infection of reproductive tissue following intravaginal inoculation was able to cross the placental barrier to infect the foetus. ZIKV has been previously shown to cross the placental barrier and infect the nascent foetus [29].

### Intravaginal-inoculated IFNAR^-/-^, wild-type C57BL/6J and guinea pigs result in foetal infection

**Fig. 3.**
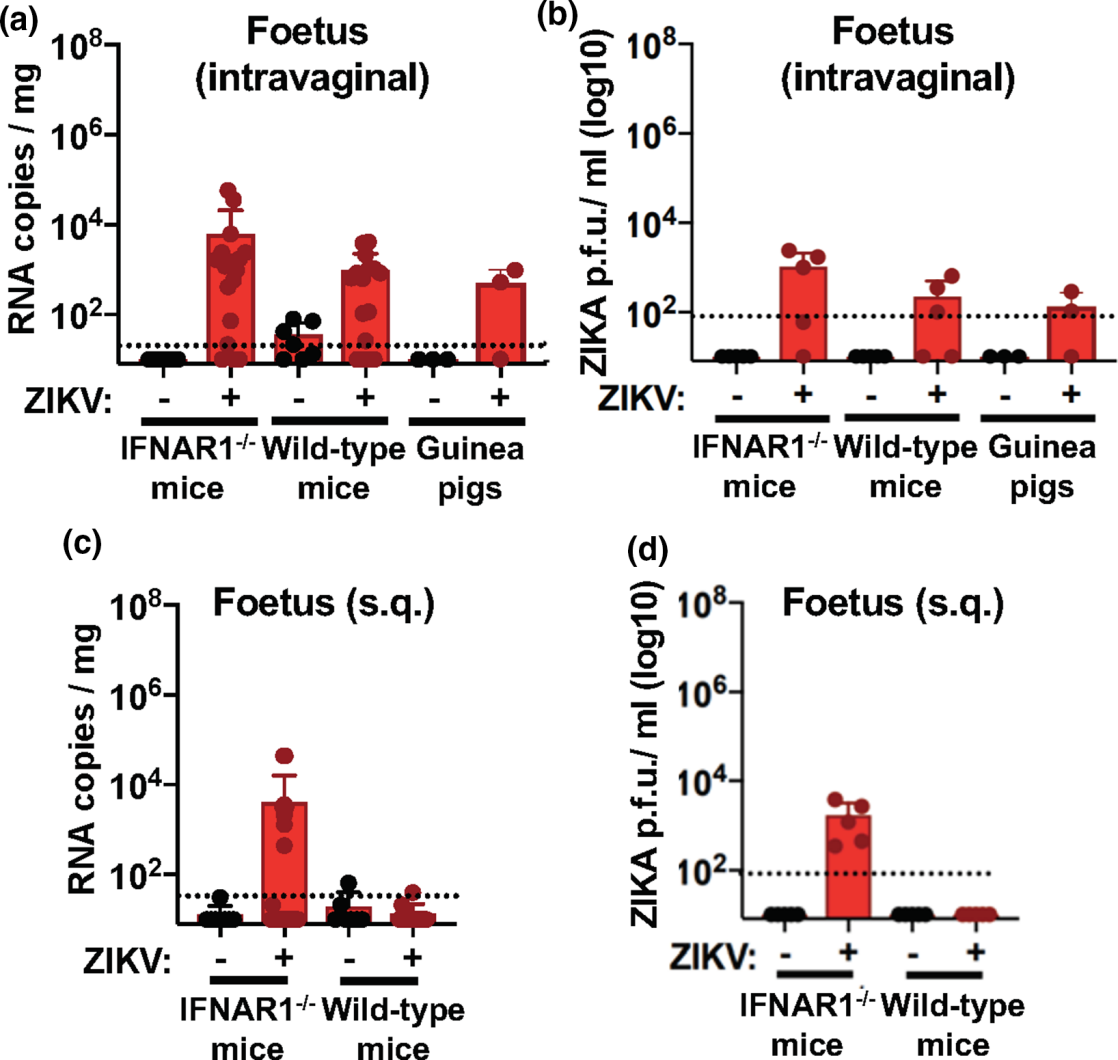
ZIKV detection in foetal tissues after intravaginal, but not subcutaneous, inoculation. Foetal tissue was evaluated at E15 (mice) or E24 (guinea pig) at 3 days p.i. Foetuses from intravaginally infected dams were evaluated for ZIKV by RT-qPCR (a) and plaque-forming assay (b). Foetuses from subcutaneous infected dams were evaluated for ZIKV by RT-qPCR (c) and plaque-forming assay (d). Data shown is representative of three experimental replicates for mice and two experimental replicates for guinea pigs (cohorts range from *n*=5–25/group for mice and *n*=3–5/group for guinea pigs).

Both RT-qPCR and plaque-forming assays performed on foetal tissue harvested from these animals demonstrated that foetuses from intravaginally inoculated IFNAR1^-/-^ (*n*=5–19/group), wild-type C57BL/6J (*n*=5–15/group) and guinea pigs (*n*=3–5/group) had detectable ZIKV (Fig. 3a, b). Histopathological evaluation by immunohistochemistry (IHC) of the placental tissues of these models revealed detection of the NS1 protein in the IFNAR1^-/-^ mice, but not in the wild-type C57BL/6J mice nor guinea pigs (Fig. S1, available in the online version of this article). Given the robust viral RNA signal detected by RT-qPCR analysis in the IFNAR1^-/-^ mice and the modest detection by IHC, the lack of detection in the immune-competent species is not unexpected. In addition to the ZIKV-specific staining, H&E staining was performed for each of the placentas evaluated and showed no clear changes across the groups (Fig. S1).

### Subcutaneous-inoculated IFNAR^-/-^ mice, but not wild-type C57BL/6J mice, result in foetal infection

In contrast, foetal tissues from subcutaneous-inoculated wild-type mice (*n*=5–13/group) were negative by RT-qPCR and plaque assay for ZIKV viral detection (Fig. 3c, d). We did however observe detectable viral RNA and replicating virus in the foetuses of subcutaneous-inoculated IFNAR1^-/-^ mice (*n*=5–25/group) (Fig. 3c, d). Taken together, these data reaffirm the high susceptibility of IFNAR1^-/-^ mice to ZIKV infections, allowing for infection of foetal tissues despite route of infection.

This illustrates that ZIKV, when inoculated intravaginally, enacts a low-level local infection that can cross the placental barrier and infect the nascent foetus in immune-competent wild-type mice and guinea pigs.

### Intravaginal ZIKV inoculation modulates gene expression profile

To promote its own proliferation and survival, ZIKV must manipulate different aspects of the host immune response. One such factor is the pro-inflammatory chemokine CXCL10. Several studies have observed ZIKV-mediated induction of CXCL10 across a spectrum of tissues and cells including within serum, monocytes, retinal epithelium, embryonic and neural progenitor cells [23, 30–33]. Elevated CXCL10 levels have been suggested as a biomarker for ZIKV infection [30]. We thus evaluated CXCL10 expression levels post-ZIKV infection to establish if these models mimic CXCL10 induction observed in human disease.

**Fig. 4.**
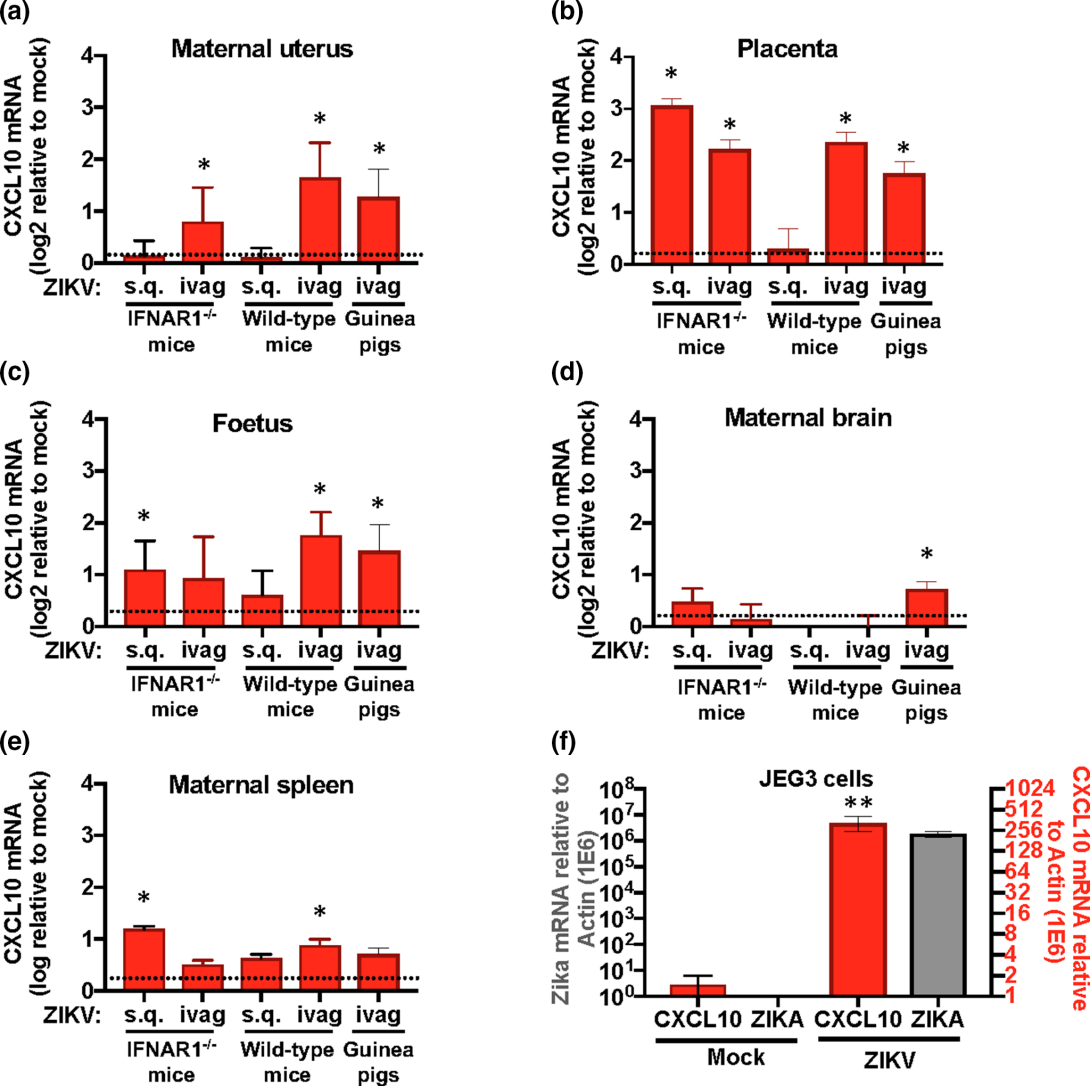
ZIKV-mediated increase of CXCL10. RT-qPCR analysis for Cxcl10 from mock or ZIKV infected pregnant IFNAR1^-/-^, wild-type mice, and guinea pigs. Uterus (a), placenta (b), foetal (c), brain (d) and spleen (e) were processed for RT-qPCR analysis of Cxcl10 transcript levels. Shown is fold change of ZIKV treated relative to mock expression levels. All transcript levels were standardized to actin expression. The human placental JEG3 cell line was infected with ZIKV at a m.o.i. of 1, and harvested after 24 h (f). Cells were processed and evaluated for ZIKV burden (grey) and CXCL10 expression (red). Statistical differences in treatment groups were determined by Student's *t*-test and * denotes significance of *P* <0.05, ** denotes significance of *P* <0.0001. Data shown is representative of three experimental replicates (cohorts range from *n*=3–5/group for mice and *n*=3–7/group for guinea pigs).

*Cxcl10* transcript levels were increased two to sixfold in the maternal uterine tissue, placenta and foetal tissue of pregnant animals (*n*=3–5/group) intravaginally inoculated with ZIKV (Fig. 4a–c). Evaluation of subcutaneous inoculated mice exhibited either no significant change or only a modest increase (less than twofold) in *Cxcl10* expression in the same tissues over mock controls (Fig. 4a–c). *Cxcl10* expression in distal tissues, such as the spleen and brain, showed only a modest increase or even a modest decrease in wild-type C57BL/6J mice and guinea pigs inoculated with ZIKV by either route (Fig. 4d, e).

To determine if the increase of CXCL10 in placental tissues mirror that observed in other ZIKV-infected human tissues, we evaluated CXCL10 transcript levels in the human placental cell line, JEG-3, infected with ZIKV (Fig. 4f). We found that CXCL10 was induced (~250-fold) in ZIKV infected cells as compared to the mock-treated controls. Taken together these findings suggest ZIKV-mediated increase of CXCL10 is conserved across several species, and lends support to the necessity for a local ZIKV infection that results in a ZIKV-specific increase of CXCL10.

### Impact of local ZIKV infection on foetal development

**Fig. 5.**
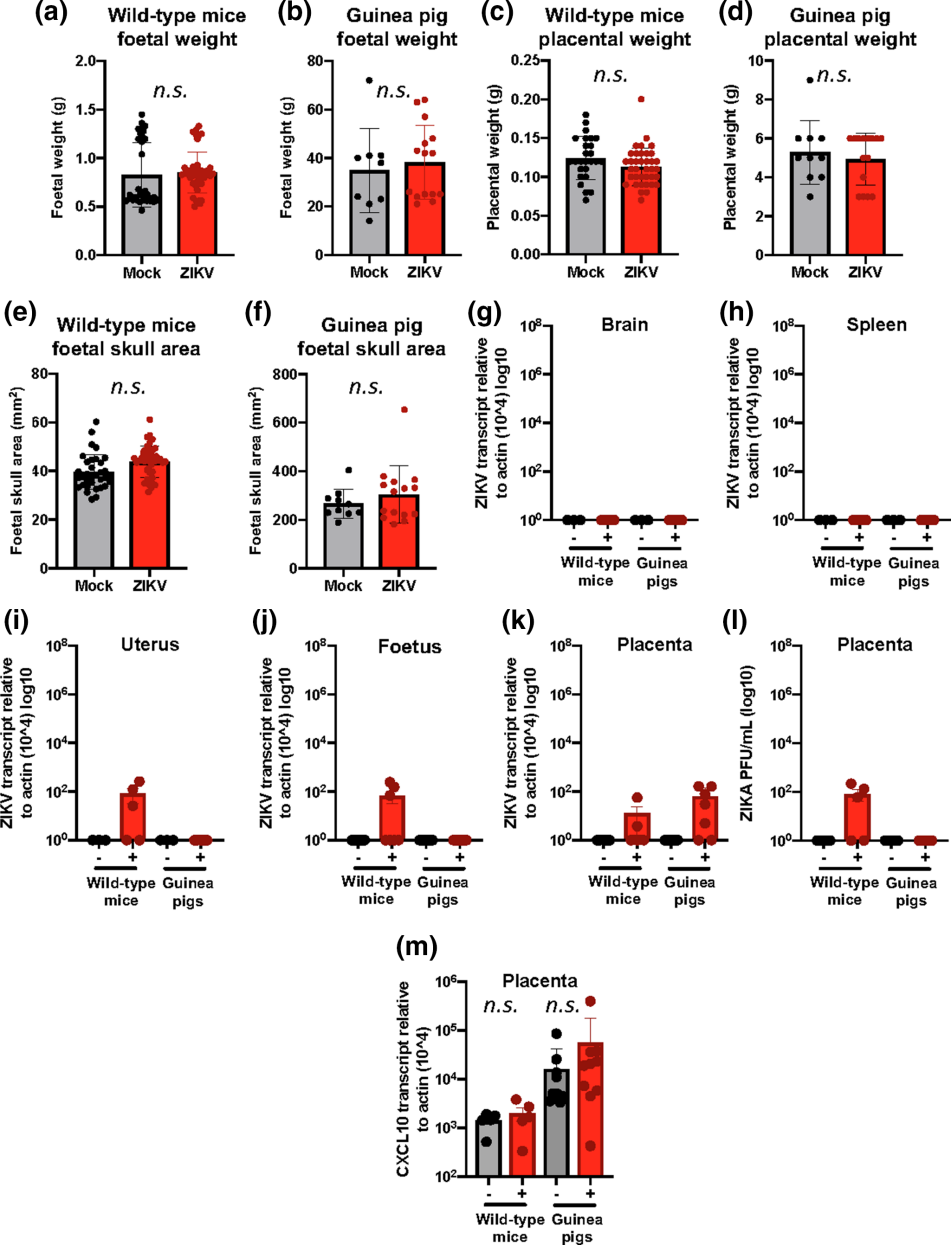
Long term foetal outcome and viral detection in ZIKV infection. Intravaginally ZIKV inoculated wildtype mice (e12–e19) and guinea pigs (e21–e58), were maintained until near parturition and sacrificed. Mouse foetal weight (a), placental weight (c) and foetal skull area (e) was determined at time of harvest. Guinea pig foetal weight (b), placental weight (d) and foetal skull area (f) was likewise evaluated. Maternal brain (g), spleen (h), uterus (i), foetal (j) and placental (k) tissues were evaluated by RT-qPCR for ZIKV burden. Placental tissues were also evaluated by plaque-forming assay for infectious virus (l). Placentas from mock and ZIKV-infected wild-type mice and guinea pigs were evaluated for *Cxcl10* transcript expression by RT-qPCR (m). *Cxcl10* transcript expression was standardized to *beta-actin* transcript expression. Statistical differences in treatment groups were determined by Student's *t*-test and *n.s.* denotes lack of significance. Data shown is representative of hree experimental replicates for mice and a single experiment for guinea pigs (cohorts range from *n*=10 dams/group with 30–45 foetal tissues for mice and *n*=6 dams/group with 10–15 foetal tissues for guinea pigs).

ZIKV infection during pregnancy is causally associated with adverse foetal outcomes. The spectrum of abnormalities varies from anatomical malformations (microcephaly, low birth weight and limb defects) to subtler intellectual and learning impairments [34]. To determine if the infections in our animal models resulted in foetal abnormalities, we infected cohorts of wild-type C57BL/-6J mice (*n*=10/group) and guinea pigs (*n*=6/group) intravaginally with ZIKV at E12 and E21, respectively. Unlike previous experiments, where the animals were euthanized 3 days p.i., these cohorts were maintained and monitored until just before parturition (E19 for mice and E58 for guinea pigs). Animals were sacrificed at this time and the foetuses were evaluated for anatomical growth abnormalities. In humans, a percentage of babies born to ZIKV-infected mothers are born underweight [35]. Evaluation of the foetal weight of both species showed non-significant differences between the mock- and ZIKV-infected animals (Fig. 5a, b). Enlarged placental weight has also shown to be an indicator of abnormal development [36]. We found no significant changes in placental weight across both treatment groups in both species (Fig. 5c, d). After the 2015–2016 outbreak, the most associated characteristic of adverse foetal outcome due to ZIKV infection is microcephaly [1]. Evaluation of the area of the foetal skull in our study showed no significant changes (Fig. 5e, f). These data suggest that although the infection was productive early/mid in the pregnancy, the severity of the infection was not sufficient to result in gross anatomical abnormalities.

It has been shown that in some tissues, ZIKV is able to be maintained for long periods of time post-infection [36]. We next determined if either maternal tissues or foetal tissues harboured ZIKV. We found the maternal brain and spleen showed no detectable viral copies in either species (Fig. 5g, h). These findings are in agreement with what was observed in the maternal tissues evaluated 3 days p.i. Interestingly**,** a minimal amount of virus was detected in the uterine and foetal tissues of wild-type mice, but not in guinea pigs (Fig. 5i, j). A possibility for this discrepancy could be the duration of gestation after inoculation. Mice have a much shorter gestation period of roughly 20 days, whereas guinea pigs have a gestation of roughly 60 days. Therefore, the duration of time post-infection to sacrifice would be 7 days for mice and 37 days for the guinea pigs. Despite this difference in time post-inoculation, we found detectable ZIKV genomes in the placentas of both species near parturition (Fig. 5k). We evaluated if these viral genomes were infectious by use of a plaque-forming assay and found that only the wild-type mouse virus was infectious, whereas the viral copies in the guinea pigs formed no discernable plaques (Fig. 5l). Despite this, long-term deposition of ZIKV in the placenta has been previously described in humans [37] and we have observed this in both pregnancy models of intravaginal inoculation.

Lastly, to evaluate if the ZIKV harboured in full-term placenta had an impact on *Cxcl10* expression, we evaluated both wild-type and guinea pig placentas for *Cxcl10* expression. We found that although there was a modest increase in *Cxcl10* in infected animals, the changes were insignificant (Fig. 5m). Although we would have expected the signal in the wild-type mouse placentas to be induced due to the presence of active virus, the timing post-inoculation may have allowed for some return back to homeostasis. Taken together, these data show that the low-level local infections initiated by intravaginal inoculation does not appear to cause gross anatomical foetal abnormalities. Despite this, it does appear that the virus can be maintained in the tissues, particularly placental, for extended periods of time.

## Discussion

Roughly 80 % of human cases of ZIKV infection are subclinical [2], and these subclinical maternal ZIKV infections can be just as detrimental to foetal health as cases presenting with clinical manifestation. Thus, animal models that exhibit few symptoms may more accurately represent what is occurring during human ZIKV infection. We explored the capacity of two immune-competent animal models, wild-type mice and Hartley guinea pigs, to determine their susceptibility to ZIKV infection using two routes of inoculation with and without pregnancy.

Here we have shown that pregnant immune-competent wild-type mice and guinea pigs intravaginally inoculated with ZIKV post-calcium-alginate swabbing [25, 38] resulted in low-level and local infections (Figs 1 and 2). Importantly these infections were able to cross the placental barrier and infect nascent foetuses (Fig. 3), in agreement with previous findings [23, 25, 38, 39]. We also demonstrate that subcutaneous ZIKV inoculation in non-pregnant and pregnant wild-type mice and guinea pigs results in inconsistent and transient infections as has been shown before [17, 18, 40]. Given the low-level circulating viral loads observed in the serum of immune-competent pregnant animals, it would be of future interest to determine if placental cells are infected [41] and/or if infection is trafficked across the placenta by immune cells [42]. Although the infections demonstrated in our models did not cause clear anatomical foetal abnormalities, it remains possible that these infections may lead to other more subtle defects as has been observed in human cases [5]. Interestingly, our studies revealed that ZIKV can be maintained long term in placental tissue post-intravaginal inoculation in both wild-type mice and guinea pigs (Fig. 5k, l). Further work will need to be undertaken to determine the activity of the virus and the consequence of its deposition [43, 44].

We demonstrated modest infections in non-pregnant wild-type mice, while others have reported variation from no clinical symptoms nor detection of virus in the animals [45, 46] to low-level circulating viral loads or localized replication in tissues [25, 47]. A common method of increasing the susceptibility to infection in wild-type mice is to utilize an IFNAR1 blocking antibody [48]. While allowing for the IFNAR1 to be maintained, a brief inhibition of the signalling allows ZIKV to replicate resulting in robust infection as compared to its untreated counterparts. Although the strength of these studies are that they allow the interferon pathways to remain intact, their temporary inhibition may not represent what occurs in natural infections. Others have used the maternal IFNAR^-/-^ crossed with a wild-type, or heterozygous, male to model maternal-foetal interactions [49, 50]. While the offspring from these crosses are reported to maintain some IFN signalling [49], the lack of the maternal immune type-1 interferon response during infection and throughout pregnancy may result in an incomplete picture of the impact of each on maternal infection and the developing foetus.

Several studies have evaluated the impact ZIKV has on pregnancy in the wild-type mouse model. The results observed are rather disparate due to the use of different virus strains and routes of inoculation. While some studies report no infection [51], others using intracranial foetal inoculations show robust deleterious effects [52, 53]. Another group reported abnormal foetal development following intravaginal ZIKV inoculation suggesting, as our data suggests, that this route of inoculation may play a key role in impacting the foetal growth environment [25]. Taken together, the immune-competent mouse model may rely heavily upon ZIKV strain, route of inoculation and gestational timing with regard to the resulting impact of low-level infections and foetal outcomes [54].

Use of the guinea pig as a model for ZIKA virus infection has been relatively limited. Our results (Fig. 1) are supported by previous work showing detectable low levels of ZIKV viral loads within the whole blood and serum of subcutaneous inoculated non-pregnant guinea pigs [9]. In contrast to our findings, this group reported ZIKV viral loads in brain and spleens of the animals as early as 2 days p.i. It is possible that we may have missed the window to detect ZIKV in the spleen and brain of our study animals (our studies terminated at 3 or 34 days p.i.), or that we inadvertently evaluated a portion of tissue not containing infection. Use of guinea pigs as a pregnant model for ZIKV has an even less well travelled history with one report showing no data with only a mention that they were unable to detect ZIKV in the pregnant model [10]. The majority of these studies incorporated subcutaneous inoculation, with a single study inoculating intravaginally in non-pregnant guinea pigs [55]. Thus, our study is the first to investigate intravaginal inoculation in a pregnancy model of guinea pigs.

The immunological environment of pregnancy is dynamic. Shifting from potently pro-inflammatory to anti-inflammatory at different stages of development to ensure implantation and healthy development. A pathogen may encounter vastly different immunological milieu depending on the stage of pregnancy [56]. It has been shown that the most susceptible time in human development to pathogenic infection is at the end of the first trimester, where the environment is highly tolerogenic and the developing foetus has not yet developed its own protective immune responses [57]. This gestational timing has been shown to be the stage that foetuses are most susceptible to congenital defects associated with ZIKV infection [58]. We have shown, in agreement with other studies [30], that the pro-inflammatory chemokine, CXCL10, is highly upregulated by ZIKV (Fig. 4). Although CXCL10 expression is necessary in early stages of development, aberrant or inappropriately timed expression has been shown to have dramatic negative impact on development [59–61]. We found that CXCL10 upregulation correlates with tissues that clear ZIKV burden. The mechanism of ZIKV-induced CXCL10 expression, which has been speculated to be associated with innate pattern recognition receptor activity [62], appears to be conserved across the different model systems from mice to humans [23, 32, 63, 64]

The long-term impact of ZIKV infection on human development is only now becoming clear. Children born to ZIKV-infected mothers during the 2016 Brazilian outbreak are presenting with a variety of associated symptoms not originally characterized [5]. These include learning and motor impairment, and deficits in their immunological capacity to combat subsequent infections. We have demonstrated infection of the developing foetuses in immune-competent wild-type mice and guinea pigs. These immune-competent animals demonstrating subclinical infection may provide insight to the mechanisms responsible for long-term deficits recognized in these children. Beyond the ZIKV field, other flaviviruses have shown the potential to cross the placental barrier to infect the foetus [65–67]. Although evidence for these infections to cause foetal disease in humans remains limited, it is worth noting that ZIKV was not associated with negative foetal outcomes until the 2015–2016 outbreak. Taken together, these studies suggest that low-level, local infections in immune-competent animals may provide more subtle information to the ZIKV field. The subclinical infections we observed in both models are more typical of those seen in the majority of human cases, and their use may play a valuable role in establishing understanding of the long-term consequences to offspring born to ZIKV-infected mothers.
