# Supplementary material for: Characterization of subclinical ZIKV infection in immune-competent guinea pigs and mice
Source: J Gen Virol. 2021 Aug 19;102(8):001641. doi: 10.1099/jgv.0.001641 (PMC8513637; doi:10.1099/jgv.0.001641)
Supplement: Supplementary material 1 [file jgv-102-1641-s001.pdf]

Supplementary Figure 1

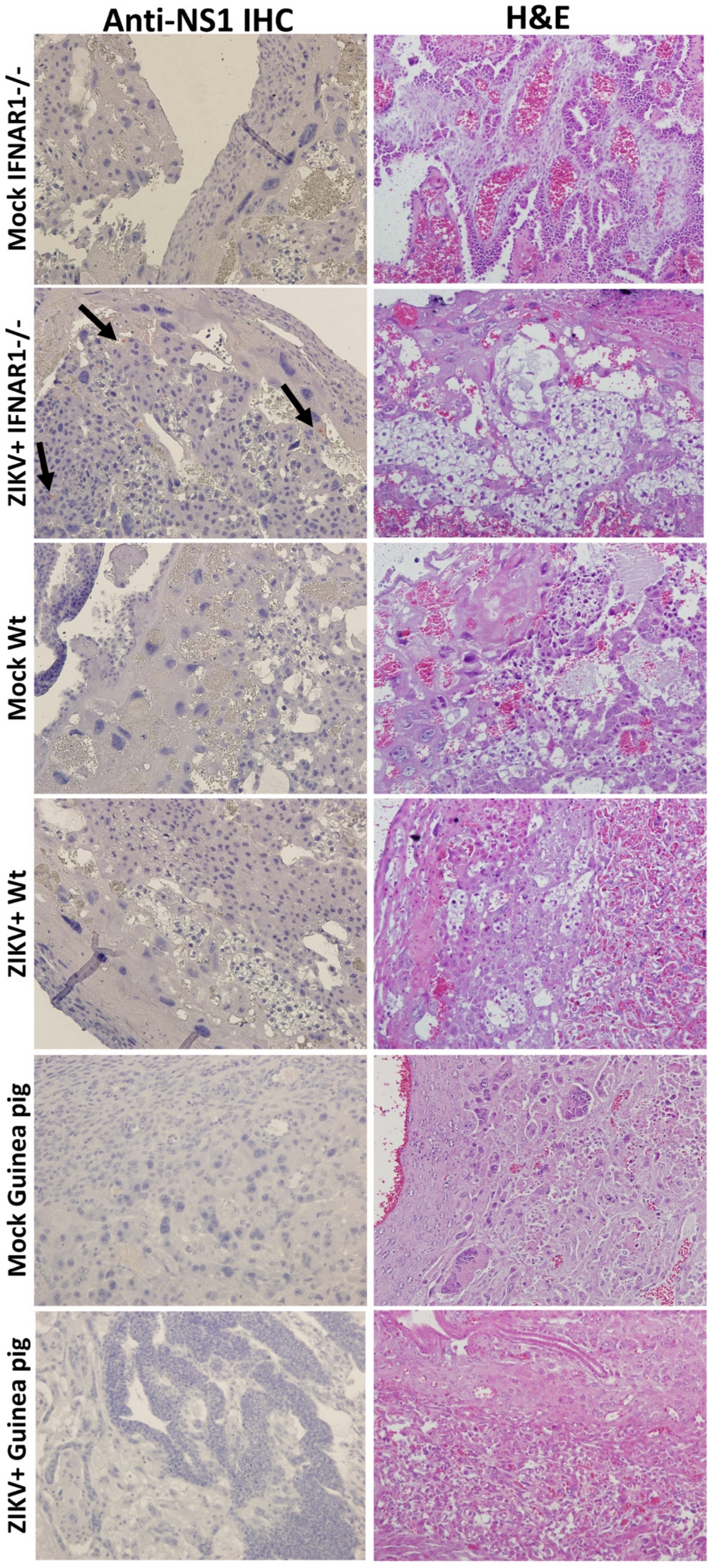

Supp. Figure 1: Histologic analysis of placenta from mock and intravaginally ZIKV inoculated pregnant IFNAR1$^{-/-}$, wildtype mice, and guinea pigs. Placentas from mock or ZIKV intravaginally inoculated (3dpi) IFNAR1$^{-/-}$, wildtype mice, and guinea pigs were probed for ZIKV using and anti-NS1 antibody (left column). Black arrows indicate positive NS1 signal. H&E staining of the same tissues is shown (right column). Shown are representative of at least 3 replicates per group.
